# Endodontic S3-level clinical practice guidelines: the European Society of Endodontology process and recommendations

**DOI:** 10.1038/s41415-025-8335-x

**Published:** 2025-04-11

**Authors:** Henry F. Duncan, Ikhlas El-Karim

**Affiliations:** 473271930612946609022 Division of Restorative Dentistry and Periodontology, Trinity College Dublin, Dublin Dental University Hospital, Dublin, Ireland; 826211503191671540381 School of Medicine Dentistry and Biomedical Sciences, Queen´s University Belfast, Belfast, United Kingdom

## Abstract

The European Society of Endodontology (ESE) published quality guidelines in 2006; however, they had not been revised since. In line with a state-of the art guideline process, the first S3-level clinical practice guideline (CPG) in endodontics was published by the ESE in 2023. The aim was to systemically develop, using a rigorous validated methodology, an S3-level guideline for the management of endodontic disease which concentrated on diagnosis and the therapeutic approaches required to deal with patients with pulpitis and apical periodontitis. Methodologically, this ESE CPG was developed with the assistance of experts from other dental disciplines and with independent methodological guidance. A systematic literature appraisal process included the analysis of comparative research in 14 specific areas that would be described in 14 systematic reviews prior to investigation of evidence's quality and strength. Finally, a series of expert and evidence-based recommendations were agreed by consensus with endodontists and a range of external stakeholders. The resulting clinical recommendations address the effectiveness of diagnosing pulpitis and apical periodontitis before recommendations are made on the effectiveness of endodontic management strategies in treatment of these diseases. Based on robust evidence, recommendations cover the effectiveness of vital pulp treatment, non-surgical and surgical management, as well as regenerative strategies to address endodontic disease. Within the guidelines, the critical importance of clinical history, asepsis, appropriate training and re-evaluation during and after treatment are underlined. In conclusion, this clinical endodontic guideline informs practice, policymakers, stakeholders and patients on the most effective treatments to manage patients with pulpitis and apical periodontitis, while highlighting critical areas for further research.

## Key points

- This review presents the clinical recommendations of the inaugural European Society of Endodontology clinical practice guideline for the management of pulpitis and apical periodontitis.
- The guidelines provide expert and evidenced-based recommendations for diagnosis and treatment in four thematic areas: treatment of pulpitis, non-surgical treatment of apical periodontitis, surgical management of apical periodontitis, and regenerative treatment of apical periodontitis.
- The process followed a robust S3-level approach, with systematic assessment of literature, independent methodological guidance, engagement with external stakeholders, including patients, and a consensus-based recommendation process.

## Background

The European Society of Endodontology (ESE) recently published inaugural S3-level clinical practice guidelines (CPG) for the treatment of pulpal and apical disease.^1^ These guidelines were principally for the benefit of clinicians, patients, researchers and other stakeholders. The ESE previously published quality guidelines for endodontic treatment in 2006;^2^ however, these needed to be updated, as the process of consensus and guideline writing had significantly changed. In the development of the current S3-level guidelines, 14 systematic reviews of the literature were undertaken before recommendations that focus on patient-centred outcomes were agreed using the Grading of Recommendations, Assessment, Development and Evaluations (GRADE) framework.^3^ This process has been employed in dentistry by the European Federation of Periodontology S3-level guidelines for treatment of stage I-III periodontitis,^4^ which have been summarised in the *British Dental Journal*.^5^

Traditionally, ESE publications including guidelines and position statements have been at the S1 level, which involves the selection of a group of experts who agree a series of recommendations based on their shared knowledge but without an official consensus process.^2^^,^^6^ For the development of a CPG, it was agreed that the development of higher-level treatment guidelines (S3) was required, which employed a structured, formalised and systematic process to ensure robustness and relevance of the findings.^7^

The ESE S3-level project was completed over two years under the auspices of a steering group, comprised of ten senior academics assigned to four working groups (WGs) designed to cover the principal facets of endodontic management: WG1 - the treatment of pulpitis; WG2 - the non-surgical treatment of apical periodontitis; WG3 - the surgical treatment of apical periodontitis; and WG4 - the regenerative treatment of apical periodontitis. The steering group worked throughout the process with the assistance of a senior, independent guideline methodologist (Ina Kopp) who advised on several issues, including methodological training, appropriate outcome measures, study selection, review process and declarations of interest. If conflicts were identified, individuals were prevented from voting and partaking in group discussion on the related subjects.

The purpose of this process was to ascertain the effectiveness of endodontic treatment for the common diseases pulpitis and apical periodontitis^8^ in a real-world practice environment, ideally evidenced by pragmatic, practice-based comparative trials. Unfortunately, as the process evolved, it became clear that the bulk of eligible studies were carried out in dental schools and universities on subpopulations and therefore measured efficacy rather than effectiveness.

In summary, this endodontic CPG makes recommendations for each diagnostic and treatment modality which can guide dental care for the general population. To that end, in this manuscript, we have summarised the recommendations using the language, ‘we recommend to', ‘we suggest to/we suggest not to' and ‘we do not know' (Table 1).^9^

**Table 1:** Strength of recommendations: grading scheme. From the German AWMF and^9^standing guidelines commission^9^

**Grade of recommendation**	**Syntax**
Strong	We recommend to (⇑⇑) We recommend not to (⇓⇓)
Weak	We suggest to (⇑) We suggest not to (⇓)
Open	We do not know/ may be considered (⇔)

## Development of clinical guidelines

The development of position statements and clinical guidelines is often hampered by personal views, bias in the selection of colleagues with similar views, conflicts of interest, and selective interpretation of data. As a result, over recent years, best-practice international standards have emerged on the most appropriate way in which to write guidelines that are robust, scientifically valid and possess minimal bias.^5^ This guideline^1^ was developed using a systemic validated approach under the auspices of methodologists who followed the guidance published by the Standing Guideline Commission of the Association of Scientific Medical Societies in Germany (AWMF)^9^ and the GRADE working group (https://www.gradeworkinggroup.org/). A decision was made in the planning stage to develop a so-called ‘evidence-to-decision' framework using a simple recommendations format (Table 1), with the evidence analysed using GRADE based on the quality of the supporting evidence and modified (up or down) by other factors, such as risk of bias, consistency, relevance and clinical effect size, balance of benefits and harm, economic and legal considerations, and finally, patient preferences and values. A major role of the steering committee is to decide the most appropriate outcomes, study types, length of follow-up and tools used for assessing the quality of studies.^10^^,^^11^

## Evidence supporting the guidelines

### Guideline development group

**Fig. 1:**
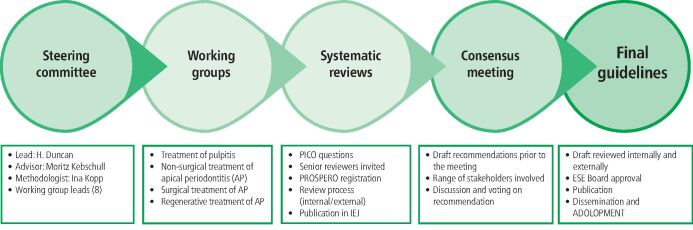
Guideline development process

The ten-member steering group met periodically online to discuss strategy and also in combination with the guideline development group, who comprised of senior academics who led the commissioned systematic reviews (SRs), relevant external stakeholders (related dental societies and organisations [i.e. related dental specialist societies, educational and research associations, International Federation of Dental Hygienists, European oral health groups], students, patient representatives) and senior endodontists. This group was tasked with evaluating the recommendations and participating in the consensus conference. Stakeholders were assigned to each of the four WGs and over a two-year period, several online plenaries and WG meetings were organised to discuss a range of issues, including process, declarations of interest, methodology, SRs, recommendations and consensus (Fig. 1). Although some WGs were larger than others, each consisted of two working group leads, two SR senior authors from each WG review, external stakeholders (chosen by invited group) and endodontists (senior, working in endodontic academia or practice, qualified greater than ten years and history of at least ten publications). WG1 had 19, WG2 had 20, WG3 had 18 and WG4 had 15 members, respectively. All votes on expert and evidenced-based recommendations were made in a plenary session, which, after declarations of interest considerations, always resulted in a voting group in excess of the quorum of 50 members voting.

### Systematic review of the literature

During initial steering group discussions, it was agreed that in order to aid comparison and reproducibility between the individual SRs, the endodontic outcome measures would have to be standardised and ranked. Furthermore, it was acknowledged that the bulk of endodontic outcomes are classically clinician-reported measures, rather than patient-reported measures, which goes against the spirit of the GRADE-based recommendation process.^12^ Therefore, before the literature search began, a list of all endodontic outcomes was compiled and ranked by the 42 members involved in the validated development process for the selecting outcomes using a nine-point Likert-scale over two rounds.^10^^,^^11^ A summary of the survey scores was shared with the members of the group before the final list of outcome measures which were rated as critical for decision-making was confirmed.^12^ For SRs addressing treatment, a combination of patient- and clinician-reported outcome measures were selected, with the most critical outcome being ‘tooth survival'. Other critical outcomes included ‘pain, tenderness, swelling, need for medication (analgesics)' and ‘evidence of resolution/emerging apical radiolucency', while additional outcomes included ‘oral health-related quality of life'.^13^ Additional outcomes were ascribed to some WGs, for example, pulpitis included ‘response to pulp sensibility test' and surgical treatment of apical periodontitis included ‘presence of sinus tract, satisfactory soft tissue healing'. Diagnostic SRs had completely different outcomes related to ‘specificity', ‘sensitivity', ‘diagnostic odds ratio', ‘positive predictive value' and the ‘negative predictive value' as probabilities for a correct test and ‘receiver operating characteristic analysis'.

At the same time, the follow-up period after treatment related to the specific outcome measure was also decided, with a minimum of one-year for assessing the effectiveness of treatments for pulpitis and apical periodontitis selected. Selected outcome measures relating to pain, swelling, medication taken, or investigating diagnostic accuracy were allocated shorter follow-up periods. The outcome measures and length of follow-up were used alongside standard instruments to assess the methodological quality of clinical trials and were then applied to all the commissioned SRs involved in the ESE S3-level guidelines.

In terms of the studies to be included, only human comparative studies with suitable follow-up were considered for inclusion.^4^ Specifically, randomised control trials and non-randomised comparative clinical trials, as well as various longitudinal observational studies, including comparative cohort and case-control studies. In order to prevent comparative case reports, there had to be at least 20 (ten in each arm) at the end of the study.

Finally, 14 SRs were commissioned by the ESE CPG steering group to evaluate the evidence in relation to focused PICOT (patient/population; intervention/indicator; compare/control; outcome; time/type) questions. The PICOT questions were standardised and homogenised by the steering group into a protocol that was submitted *a priori* to the PROSPERO database. Later, all 14 SRs were published in the *International Endodontic Journal* after a rigorous peer-review process, including internal and external review.^14^^,^^15^^,^^16^^,^^17^^,^^18^^,^^19^^,^^20^^,^^21^^,^^22^^,^^23^^,^^24^^,^^25^^,^^26^^,^^27^ In all the SRs, the risk of bias was evaluated using tools specific to each study type (Table 2), with Cochrane's RoB 2 for randomised controlled trials, the Newcastle-Ottawa scale for observational studies, and QUADAS-2 for studies investing diagnostic efficacy.

**Table 2:** Risk of bias assessment tools

**Study design**	**Risk of bias tools**
Randomised control trials	RoB2 https://methods.cochrane.org/bias/resources/rob-2-revised-cochrane-risk-bias-tool-randomized-trials
Controlled clinical trials (non-randomised)	ROBINS-I https://methods.cochrane.org/robins-i
Comparative cohort, case control	Newcastle Ottawa Scale for observational studies https://www.ohri.ca/programs/clinical_epidemiology/oxford.asp
Diagnostic accuracy studies	QUADAS-2 https://www.bristol.ac.uk/population-health-sciences/projects/quadas/quadas-2/

## Structured consensus summit

The structured consensus summit for ESE S3-level treatment of pulpal and apical disease was held in Lisbon, Portugal on 29 January to 1 February 2023. Using the 14 SRs, pre-prepared evidence-based recommendations were debated by the guideline development panel using a structured consensus development format under the leadership of an independent methodologist. At the summit, WG discussions and open plenary discussions were timetabled, where the proposed recommendations were presented, voted upon and adopted by consensus.^28^ As described previously, delegates declaring potential conflicts abstained from voting or discussion.

## Key clinical recommendations

The CPG encompasses 34 key clinical recommendations - ten expert-based and 24 evidenced-based - for the diagnosis and management of pulpitis and apical periodontitis. Below, a selection of the main recommendations are summarised. For a detailed description of the method, process and recommendations, please refer to the original publication.^1^

### General expert-based recommendations

Pulpitis may be accompanied by pain and sensitivity associated with a tooth, while apical periodontitis may be symptomatic or asymptomatic, generally presenting with radiographic evidence of an apical radiolucency. These diseases are principally caused by bacterial challenge and can impact on the patient's quality of life and necessitate extraction treatment if appropriate and timely endodontic treatment is not carried out. The fundamentals of high-quality care are often not investigated in clinical trials (e.g. dental dam use); however, these factors underpin the practice of endodontics. For that reason, expert-based recommendations were used to stress the essential components of endodontic management:

- ‘For the management of restorable teeth with pulpitis, it was recommended that either vital pulp treatment or root canal treatment, appropriate restoration to function and supportive postoperative care, were preferable to extraction'
- Likewise, ‘for the management of restorable teeth with apical periodontitis*,* it was recommended that root canal treatment, appropriate restoration and supportive postoperative care, rather than extraction, was preferable'
- To limit the prescription of antibiotics in endodontics,^29^ it was recommended that for ‘the emergency management of symptomatic pulpitis or apical periodontitis in a restorable tooth, vital pulp treatment or root canal treatment, rather than extraction or systemic antibiotic prescription, was appropriate'
- In order to encourage good practice in endodontics and ensure that the evidence supporting these guidelines^1^^,^^2^^,^^15^ translates to practice for the non-surgical management of the exposed pulp exposure, as well as pulpal and apical disease, it was recommended that ‘a meticulous aseptic technique and optimal surgical field, including the use of dental dam, good light and magnifying devices should be used'
- With respect to the appropriate postgraduate training required to carry out endodontic treatment, existing recommendations^30^^,^^31^ and respecting a broad range of standards globally, we suggested that for advanced non-surgical techniques including ‘complex retreatment and for surgical management of apical periodontitis, further postgraduate training would be beneficial'. Although complex retreatment was not defined in the CPG, according to the American Association of Endodontists' case difficulty assessment, high-difficulty cases would generally relate to multi-rooted teeth, or teeth with intracanal obstructions, such as fractured files, posts, ledges or perforations^32^
- Finally, in term of monitoring the success of treatment,^2^^,^^6^ ‘after vital pulp treatment to manage pulpitis or non-surgical or surgical treatment of apical periodontitis, it was recommended that cases are monitored for a prolonged period with the review period extended if there is uncertainty about healing'. The exact time period was not stipulated due to absence of firm data suggesting that stating any specific number of years was evidenced-based.

### Evidenced-based recommendations relating to diagnosis

In order to manage endodontic disease appropriately, current diagnostic methods were analysed in their ability to diagnose the presence or absence and severity of disease. The diagnosis of pulpitis^17^ and apical periodontitis^18^ were both investigated:

- When ascertaining a diagnosis of pulpitis in patients suspected of having pulpitis with either no pain, non-spontaneous pain or spontaneous pain, ‘we suggested the use of cold testing possibly supplemented by electric pulp testing to assess pulp vitality. Furthermore, we suggested a combination of pain history (presence of pain, history of previous pain and occurrence of spontaneous pain) with clinical conditions (presence of pulp exposure, tenderness to percussion and pain on heat stimuli) to assess pulpal condition. We also recognised that we did not know whether inflammatory biomarkers can predict the inflammatory status of the pulp, highlighting an area for future research activity'
- ‘In patients suspected of having apical periodontitis, we strongly recommended periapical radiography be routinely used to diagnose apical periodontitis and that CBCT (cone beam computed tomography) may be considered as an additional diagnostic measure, in cases where there is doubt about the diagnosis. Notable is the presence of radiopaque materials in the root canal and periapex may affect the diagnostic accuracy of CBCT'.^18^

### Evidenced-based recommendations relating to the treatment of pulpitis

The effectiveness of vital pulp treatment in managing pulpitis with no or non-spontaneous pain,^19^ spontaneous pain,^26^ as well as the effectiveness of root canal treatment in managing teeth with vital or necrotic pulps,^24^ was investigated (Table 3):

**Table 3:** Key recommendations - vital pulp treatment and revitalisation

**Intervention**	**Grade and evidence-based recommendations**	**Expert consensus-based recommendations**
Selective caries removal versus direct pulp capping or pulpotomy for non-traumatic pulpitis associated with no or no spontaneous pain	No studies identified	Either selective caries removal without pulp exposure or after pulp exposure direct pulp capping or pulpotomy may be considered
Direct pulp capping versus pulpotomy for non-traumatic pulpitis associated with no or no spontaneous pain	Open (⇔) Either direct pulp capping or pulpotomy (partial/full) may be considered Quality of evidence: Very low ⊕⊝⊝⊝	
Pulpotomy versus pulpectomy for non-traumatic pulpitis associated with no or no spontaneous pain	Open (⇔) Either full pulpotomy or pulpectomy may be considered Quality of evidence: Very low ⊕⊝⊝⊝	
Pulpotomy versus pulpectomy for non-traumatic pulpitis associated with spontaneous pain	Weak (⇑) We suggest treatment with either root canal treatment or full pulpotomy Quality of evidence: Low ⊕⊕⊝⊝	
Treatment of pulp necrosis with or without apical periodontitis in immature permanent teeth	Open (⇔) The apical plug technique or revitalisation procedures may be considered Quality of evidence: Low ⊕⊕⊝⊝	
Treatment of pulp necrosis with or without apical periodontitis in mature permanent teeth with revitalisation procedures	Weak (⇓) We suggest not to use revitalisation procedures Quality of evidence: Low ⊕⊕⊝⊝	
Endodontic tissue engineering in immature permanent teeth	Open (⇔) We do not know whether endodontic tissue engineering represents a valid treatment option. Further research is necessary to address this lack of evidence

- ‘Firstly, in patients with pulpitis associated with no or non-spontaneous pain in permanent teeth, we do not know whether direct pulp capping or pulpotomy (partial/full) is as effective as selective or stepwise caries removal regarding the long-term survival of the pulp or the tooth'. This evidence-based recommendation highlighted the lack of studies focused on this question, which could be attributed to ethical issues, as well as the practicality of organising such a study.^33^ As a result of an absence of studies, expert-based recommendations were made, including that the use of either selective/stepwise caries removal without pulp exposure or after pulp exposure direct pulp capping or pulpotomy (partial/full) may be considered. Furthermore, if the pulp was exposed, an enhanced protocol was recommended for subsequent vital pulp therapy, which consists of the use of dental dam, antimicrobial lavage, magnification, and use of a hydraulic calcium silicate cement (HCSC). From an evidenced-based perspective, for pulpitis in the absence of symptoms or non-spontaneous pain, either direct pulp capping or pulpotomy (partial/full) may be considered. Full pulpotomy and pulpectomy based on the evidence may also be considered.
- If the pulpitis was associated with spontaneous pain in permanent teeth based on the evidence,^26^ ‘we suggested either root canal treatment or full pulpotomy could be effective'
- No difference was shown between vital and non-vital teeth in terms of outcome measures; however, ‘we suggested that root canal treatment is performed on teeth with non-vital pulps as soon as the diagnosis is confirmed'.^24^

### Evidenced-based recommendations relating to the treatment of immature teeth with apical periodontitis

The management of immature teeth differs in relation to the treatment of apical periodontitis, as conventional root canal preparation and filling is not possible using conventional techniques:

- ‘For patients with immature permanent teeth and pulp necrosis ± apical periodontitis, the HCSC apical plug technique or revitalisation procedures may be considered'^21^
- ‘At present, we do not know whether endodontic tissue engineering represents a valid treatment option as further clinical evidence and research is required'.^27^

### Evidenced-based recommendations relating to the non-surgical treatment of mature teeth with apical periodontitis

When considering the non-surgical treatment of apical periodontitis in mature teeth, various aspects of management were considered, including root canal instrumentation,^15^ dressing,^25^ irrigation,^25^ filling^22^ and adjunct aids^20^ (Table 4):

**Table 4:** Key recommendation - non-surgical treatment of apical periodontitis

**Intervention**	**Grade and evidence-based recommendations**
Instrumentation performed with contemporary techniques versus ‘traditional' stainless-steel instruments technique	Weak (⇑) We suggest root canal preparation should be performed using contemporary engine-driven techniques with NiTi root canal instruments Quality of the evidence: Survival, postoperative pain: Moderate ⊕⊕⊕⊝ Radiographic healing: Low ⊕⊕⊝⊝
Irrigation with any root canal irrigant(s) and sequence versus irrigation with NaOCl and EDTA	Open (⇔) NaOCl (1-5.25%) followed by EDTA, and NaOCl (1-5.25%) may be considered Quality of evidence: Postoperative pain and radiographic healing: Very low ⊕⊝⊝⊝
Intracanal dressing with any root canal dressing(s) versus calcium hydroxide versus no dressing	Strong (⇑⇑) Where adequate clinical procedures have been performed, we recommend using a single-visit approach without the use of inter-appointment calcium hydroxide Quality of evidence: Radiographic healing: Moderate ⊕⊕⊕⊝
Root canal filling with any other type of sealer versus epoxy resin (AH Plus/AH 26) using gutta-percha	Open (⇔) Root canal filling with gutta-percha in combination with any of the included sealers (epoxy resin, ZOE or calcium silicate) may be considered
Root canal filling with any type of non-lateral compaction technique versus cold lateral compaction technique using gutta-percha	Open (⇔) Root canal filling with gutta-percha and sealer using any of the included techniques (cold lateral compaction, warm vertical compaction, carrier based or single cone) may be considered
Adjunct therapy versus traditional syringe and needle irrigants delivery	Weak (⇓) We suggest not to use adjunct therapy in addition to traditionally (syringe-needle-based) delivered irrigants Quality of evidence: Pain and radiographic healing Low ⊕⊕⊝⊝

- ‘In terms of instrumentation, for patients with apical periodontitis, any tested type of engine-driven nickel titanium (NiTi) instruments may be considered for root canal preparation, as there was no evidence suggestive of an advantage of one particular file system over another'^15^
- For irrigation, in patients with asymptomatic apical periodontitis in permanent teeth, an open recommendation of sodium hypochlorite (NaOCl) (1−5.25%) followed by ethylenediaminetetraacetic acid (EDTA), and NaOCl (1−5.25%) may be considered
- With respect to interim dressing, a strong recommendation was made that where adequate clinical procedures had been performed, a single-visit approach without the use of inter appointment calcium hydroxide should be adopted^25^
- ‘Root canal filling with gutta-percha and sealer using any of the included techniques (cold lateral compaction, warm vertical compaction, carrier-based or single cone) may be considered in combination with any of the included sealers (epoxy-resin, ZOE or calcium-silicate)'
- ‘In patients with apical periodontitis, we suggest not to use adjunct therapy in addition to traditionally (syringe-needle based) delivered irrigants. Adjunct therapy would include photoactivated disinfection, direct laser irradiation, ozone therapy and passive ultrasonic activation. At present, there is not comparative outcome evidence to indicate that these are more effective than conventional techniques'
- Finally, in patients with pulp necrosis in mature permanent teeth, ‘it was suggested not to use revitalisation techniques. This was in contrast to the recommendations made for immature permanent teeth'.

### Evidenced-based recommendations relating to the surgical treatment of mature teeth with apical periodontitis

For surgical treatment of apical periodontitis in mature teeth, various aspects were considered, including comparison of surgery with non-surgical root canal treatment/retreatment,^14^ effectiveness of root resection^16^ and the effectiveness of intentional reimplantation (Table 5):^23^

**Table 5:** Key recommendations - surgical treatment of apical periodontitis

**Intervention**	**Grade and evidence-based recommendations**	**Expert consensus-based recommendations**
Surgical versus non-surgical root canal treatment/retreatment	Open (⇔) When non-surgical root canal treatment or retreatment is impractical, apical surgery may be considered for the management of permanent teeth with apical periodontitis Quality of evidence: Tooth survival: Low ⊕⊕⊝⊝	
Root resection versus non-surgical root canal retreatment or apical surgery	Weak (⇓) We do not suggest root resection techniques as an alternative to non-surgical root canal retreatment or apical surgery in the management of permanent teeth with apical periodontitis Quality of evidence: Tooth survival: Very low ⊕⊝⊝⊝	
Intentional replantation versus non-surgical root canal treatment/retreatment or apical surgery	Weak (⇓) We do not suggest intentional tooth replantation as a routine alternative to nonsurgical root canal treatment/retreatment or apical surgery for managing permanent teeth with apical periodontitis Quality of evidence: No comparative studies identified	Non-comparative clinical studies reported high overall survival rates in the mid- to long-term, with relatively low complication rates. Therefore, in the absence of other treatment alternatives and rather than extraction, if anatomical conditions permit atraumatic extraction and an extra-oral time of less than 15 minutes, then intentional replantation may be considered for the management of permanent teeth with apical periodontitis

- ‘The evidence did not suggest a difference in the effectiveness of apical surgery, compared with non-surgical root canal treatment or retreatment, in terms of clinical, radiological, and patient-related outcomes, for managing permanent teeth with apical periodontitis. Therefore, when non-surgical root canal treatment or retreatment are impractical, apical surgery may be considered for the management of permanent teeth with apical periodontitis'^14^
- Due to a lack of comparative evidence, ‘root resection and intentional replantation techniques were not suggested as an alternative to non-surgical root canal retreatment or apical surgery in the management of permanent teeth with apical periodontitis. However, due to the presence of non-comparative data supporting its use intentional replantation procedures could be considered as a clinical option'.^16^^,^^23^

## Implementation, dissemination and update

Further to the publication of the open-access CPG accompanied by 14 SRs in a special issue of the *International Endodontic Journal*, a layered communication plan was ratified by the ESE Executive Board and delegated to the ESE Benefits of Endodontics Committee. This included:

1. A programme of adoption and adaptation^34^ by 37 ESE national member societies, including translation into selected languages, including French, Italian, Spanish, German, Chinese and Polish
2. Dissemination of the findings in designated symposia sessions at ESE conferences (Helsinki 2023 and Krakow 2024)
3. Publication of simple ‘bite-sized' outputs from the guidelines through the ESE communications committee. As the CPG is an evidenced-based document that will need to be updated as new evidence emerges, it is planned that the guideline is valid for five years until at least 2028.

## Perspectives and summary

The CPG is the first comprehensive guideline for the diagnosis and treatment of pulpitis and apical periodontitis that follows the structured S3-level procedure. It covers the principal components of endodontic treatment but does not cover all aspects of endodontic care, including periodontal endodontic lesions, vertical root fractures, dental traumatic injuries and resorption, among others. It is hoped that these will be covered in future S3-level projects or in traditional position statement format.

The recommendations not only list current evidenced-based knowledge in the discipline but also highlights gaps in knowledge and areas for further priority and research. The next steps will be to engage in a period of activity to disseminate the findings of these guidelines to endodontists, dentists, and relevant stakeholders, including students and patients.
